# Master regulatory role of p63 in epidermal development and disease

**DOI:** 10.1007/s00018-017-2701-z

**Published:** 2017-11-04

**Authors:** Eduardo Soares, Huiqing Zhou

**Affiliations:** 1 Department of Molecular Developmental Biology, Faculty of Science, Radboud Institute for Molecular Life Sciences, Radboud University, 274, Postbus 9101, 6500HB Nijmegen, The Netherlands; 2 CAPES Foundation, Ministry of Education of Brazil, Brasília, Brazil; 3 Department of Human Genetics, Radboud University Medical Center, 855, Postbus 9101, 6500HB Nijmegen, The Netherlands

**Keywords:** Epidermis, Gene regulation, Ectodermal dysplasia, Epidermal cell identity, Epigenetics

## Abstract

The transcription factor p63 is a master regulator of epidermal development. Mutations in p63 give rise to human developmental diseases that often manifest epidermal defects. In this review, we summarize major p63 isoforms identified so far and p63 mutation-associated human diseases that show epidermal defects. We discuss key roles of p63 in epidermal keratinocyte proliferation and differentiation, emphasizing its master regulatory control of the gene expression pattern and epigenetic landscape that define epidermal fate. We subsequently review the essential function of p63 during epidermal commitment and transdifferentiation towards epithelial lineages, highlighting the notion that p63 is the guardian of the epithelial lineage. Finally, we discuss current therapeutic development strategies for p63 mutation-associated diseases. Our review proposes future directions for dissecting p63-controlled mechanisms in normal and diseased epidermal development and for developing therapeutic options.

## Introduction

The transcription factor p63, encoded by the *TP63* gene, belongs to the p53 gene family. Distinct from the leading member of the gene family, p53, that plays an important role in tumor suppression, the role of p63 in cancer is not fully understood. However, p63 has been shown to be a key regulator of epidermal development. This has been demonstrated in various animal models and by p63 mutation-associated human diseases. For example, complete deletion of p63 in mice results in the absence of the epidermis and epidermal related appendages, as well as defects in other epithelial-related tissues [1–3]. In humans, heterozygous mutations in *TP63* cause several developmental disorders, and many of these diseases manifest skin abnormalities [4–7]. Classical studies showed that p63 is an important player in embryonic epidermal development and in epidermal keratinocyte proliferation and differentiation, where it directly regulates numerous target genes involved in cell proliferation, differentiation and adhesion [8–10]. Several recent studies demonstrated that p63 also plays a role in the modulation of the epigenetic and chromatin landscape in epidermal keratinocytes by directly regulating chromatin factors and by engaging and opening chromatin regions [11–16]. Among these studies, those using unbiased genome-wide approaches convincingly established that p63 is a key regulator controlling the enhancer landscape. These recent findings suggest a more sophisticated model of the master regulatory role of p63 and reveal additional layers of complexity in p63-orchestrated gene regulation of epidermal development and related diseases.

Different p63 isoforms have been shown to play roles in various cells and tissues, such as the epidermis, oocytes, muscles and cochlea. As such, this review discusses p63 isoforms identified so far, with a focus on isoforms that are expressed in epidermal cells. Subsequently, we dedicate most of this review to the most studied topic in p63 biology: the role of p63 in epidermal development and related diseases. Specifically, we provide an overview of p63 mutation-associated developmental diseases with epidermal phenotypes. We discuss in-depth key molecular and cellular mechanisms by which p63 controls gene regulation to define epidermal identity, and speculate that the affected cell fate contributes to various disease states. Finally, we review current efforts and future perspectives in developing therapeutic strategies for treating these diseases.

## p63 isoforms and their expression

**Fig. 1:**
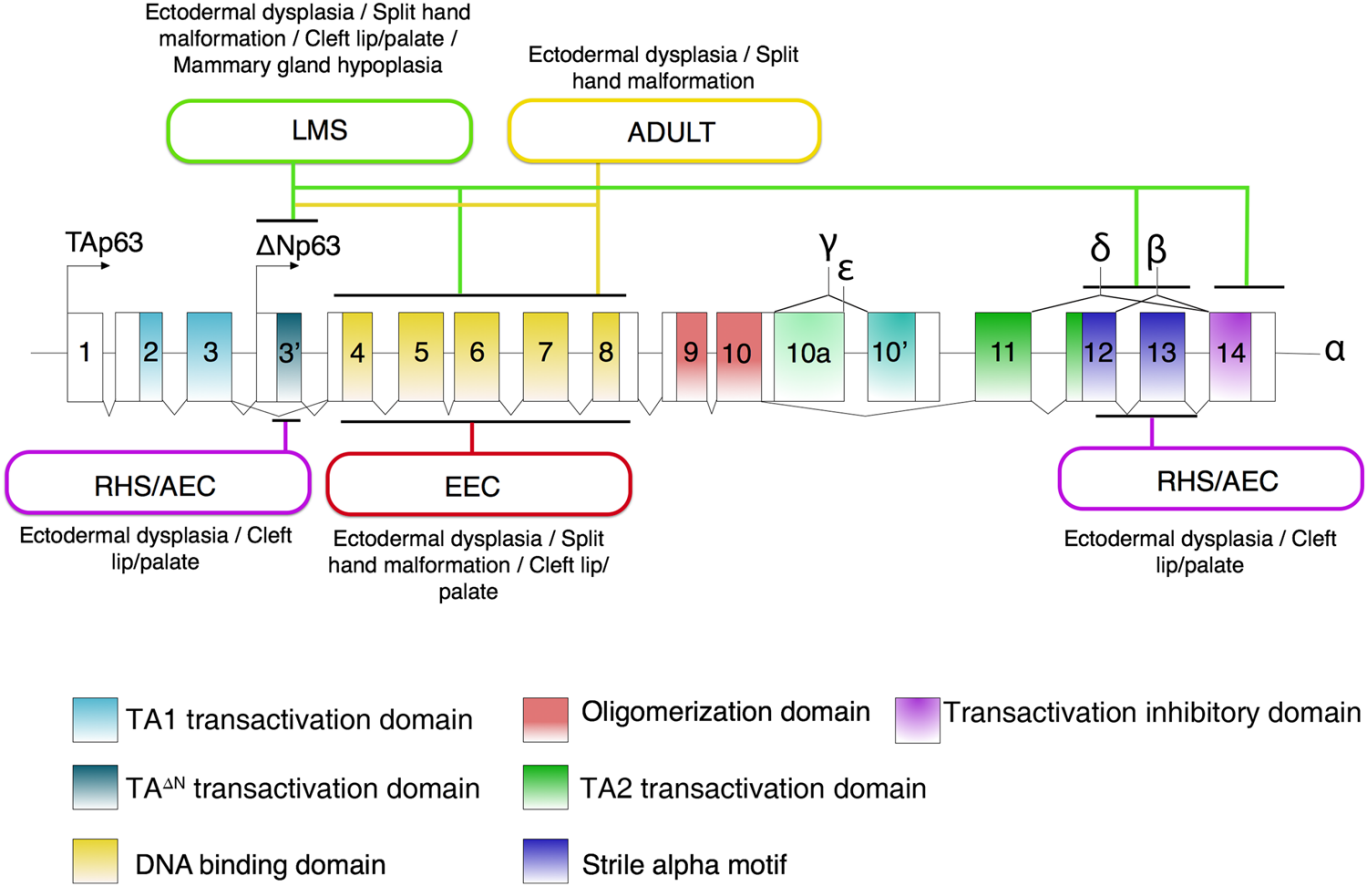
Gene and protein structures of p63 and mutations involved in developmental syndromes with epidermal phenotypes (ectodermal dysplasia). Two promoters resulting in N-terminal TA and ΔN isoforms are indicated with arrows. Exons and protein domains are numbered and color-coded as indicated. p63 mutation-associated ectodermal dysplasia syndromes are marked in round-edge rectangles and the locations of their associated hotspot mutations are indicated by black lines. The main phenotypes related to the syndromes are shown near the rectangles

The transcription factor p63 was initially described as keratinocyte transcription factor (KET) in 1997, as it is homologous to p53 in rat epithelial tissues [17]. One year later, it was denoted as one of the p53 gene family members in a comprehensive cloning study [2]. In this study, six isoforms resulting from two alternative promoters (TA and ΔN) and three different splicing routes (α, β, γ) were identified from mouse E15 embryos and a human neuroepithelioma cell line [2], indicating that these isoforms were expressed in those cells and tissues. The TA isoform contains three TA-specific exons, exon 1, 2 and 3, and encodes a transactivation domain (TA1) that is homologous to the transactivation domain of the p53 protein (Fig. 1). Another promoter is used to produce a shorter isoform denoted ΔN, and its starting exon, named as exon 3′, is specific to the ΔN isoform. Initially, the ΔN isoform was considered transcriptionally inactive, functioning as a dominant negative variant towards the TA isoform, because it lacks the typical transactivation domain TA1. However, it has since been recognized that the N-terminal region of the ΔN isoform also possesses transactivation activity, and therefore it is termed as TA^ΔN^ (Fig. 1). At the C-terminus, the longest isoform is the α isoform that contains all 3′ exons 11–14 (Fig. 1). At the protein level, the α isoform contains a sterile alpha-motif (SAM) domain that is thought to be involved in protein–protein interactions [2] and a Transactivation Inhibitory Domain (TID) that inhibits the activity of the TA1 domain [18]. Two other isoforms, β that lacks the exon 13 and γ that does not have exons 11–14 but has a γ-specific exon 10′, do not contain the SAM and TID domains.

In recent years, several novel isoforms have been reported. At the N-terminal end of the p63 protein, an alternative translational start site located in the 4th exon gives rise to a ΔΔN isoform that lacks the first 26 amino acids of the ΔN isoform in epidermal keratinocytes (Fig. 1). This isoform was identified by Rinne et al. [19]. In their study on AEC/RHS syndrome, stop mutations were found within the first 26 amino acids of p63 in several AEC/RHS patients. To the surprise of the authors, the *TP63* transcript was detected, rather than being degraded completely via the nonsense-mediated decay mechanism that is common for stop mutations. This incidental observation led to the identification of an alternative translational start site downstream of all stop mutations. This alternative translational start site gives rise to the ΔΔN protein variant in AEC/RHS patient keratinocytes, and it is also present in keratinocytes of healthy controls, although at a lower level than the ΔN isoform. At the C-terminal end, two other isoforms, δ and ε, were identified by bioinformatic analyses using the Alternative Splicing Prediction Data Base, ASPicDB (http://www.caspur.it/ASPicDB/index.php) and validated by reverse transcription quantitative PCR (RT-qPCR) in several cell lines including keratinocytes, HEK293 cells and in some tissues, such as the muscle and the brain [20]. The δ isoform is an alternatively spliced variant that lacks exon 13. The ε isoform is generated by a premature stop codon that is located in exon 10. These isoforms were later validated by an RNA-seq analysis in mouse keratinocytes [21]. In the same mouse keratinocyte RNA-seq study, the authors additionally identified a slightly shorter isoform that lacks four amino acids encoded by nucleotides located in exon 8. Furthermore, in a p63 isoform analysis using RNA-seq data from squamous cell carcinoma (SCC) cell lines and from publically available data from cells of all three germ layer origins, Sethi et al. confirmed all previously reported p63 isoforms in different cell types [22]. Isoforms identified so far all contain the DNA-binding domain (DBD) and the oligomerization domain (OD) (Fig. 1).

Expression patterns and functions of TA and ΔN isoforms during epidermal commitment and subsequent stratification have been under debate, although it is generally agreed that the ΔN isoform is highly expressed in basal epithelial cells. One report showed that the TA isoform was expressed first and subsequently required to initiate epidermal stratification, but it was counter-balanced by the ΔN isoform during stratification [23]. In contrast, another study showed the ΔN isoform was required for the proliferation of epidermal cells in the basal layer, while the TA isoform was required for activating genes during stratification [24]. These studies proposed an interesting switch of the two isoforms during epidermal development. However, these observations have not been confirmed by other studies. The majority of current literature agrees that the ΔN isoform is the major functional isoform in epithelial cells and tissues, such as oral and dental tissues, corneal tissues and lung epithelial cells [1, 3, 25–28]. This scenario is supported by phenotypes presented in ΔNp63-specific knockout mouse models that, similar to mice that lack all p63 isoforms, showed defects of the skin, oral epithelium, mammary glands, limb and craniofacial regions [27].

As recently developed genomic approaches allow detecting active promoters and expressed exons, these techniques greatly facilitate the identification of isoform expression at the transcript level. Several independent analyses using RNA-seq, ChIP-seq of RNA polymerase II, DNA hypersensitivity sites (DHS)-seq and cap analysis gene expression (CAGE) [14, 22, 29] showed that the ΔN promoter is the only active promoter and the 3′ exon is the first expressed exon detected in epidermal cells throughout epidermal stratification. Furthermore, in line with the current literature mentioned above, Sethi et al. reported that the ΔN isoform is the only abundantly expressed isoform in many epithelial cells, such as those from oral tissues and the mammary gland among 40 human cell types [22]. In contrast to the abundant expression of the ΔN isoform in epithelial cells, the TA isoform is generally expressed at a low level in a range of non-epithelial cells. It is expressed at a low to moderate level in Burkitt Lymphomas (BL) cell lines and the lymphoblastoid cell line GM12878 [22]. Although the findings from novel genomic analyses [14, 22, 29] do not exclude the possibility that the TA isoform is expressed at a very low level below the detection threshold, they do cast doubts on the importance of the TA isoform is in epithelial cells, given that its expression is at least several magnitude lower than the ΔN isoform. Interestingly, the TA isoform has been shown to play roles in various organs and tissues other than the epidermis. TAp63 is expressed in oocytes and plays an important role in controlling apoptosis in response to DNA damage [28]. In the cochlea, the TA isoform is also expressed and regulates the Notch signaling pathway, which is required for proper cochlea development [30]. Additionally, TAp63 has been found to be expressed in late-stage myogenesis [31] and in cardiomyocyte development [32]. In general, the TA isoform seems to play a role in stress- or condition-induced response and in senescence, aging and metabolism [20, 27–30]. Many of these reported functions of the TA isoform are consistent with phenotypes observed in TA-specific mouse models [33–35], and accordingly, these phenotypes of TA-specific mouse models are distinct from the strong epidermal, orofacial and limb phenotypes of the p63 ΔN-specific or complete knockout mouse models [1, 3, 27].

As for p63 C-terminal isoforms, the α isoform is the predominant one in most p63-expressing cells [22], although β, δ and ε isoforms are also expressed at a low level [20, 22]. The γ isoform is reported to be expressed in muscle cells [20, 31], immortalized cancer cells, such as MCF7 [20] and squamous cell carcinoma cell lines [22]. As the α isoform is by far the most abundant isoform in cells detected so far, it has been proposed that the bulk of the p63 functional activity is driven by the α isoform [22]. Further biochemical and functional in vivo analyses of other isoforms will be important for dissecting their function.

## p63 mutation-associated diseases with ectodermal dysplasia

Although p63 isoforms are expressed in a range of tissues of different germ layer origins, germline mutations of *TP63* have until now only been associated with ectodermal-related disorders manifested with three hallmark defects: ectodermal dysplasia, limb malformation and orofacial clefting [5]. These diseases include Ectrodactyly, Ectodermal Dysplasia, and Cleft lip/palate syndrome (EEC, OMIM 604292), Ankyloblepharon-Ectodermal defects-Cleft lip/palate (AEC, OMIM 106260), Limb Mammary Syndrome (LMS, OMIM 603543), Acro-Dermato-Ungual-Lacrimal-Tooth syndrome (ADULT, OMIM 103285), Rapp–Hodgkin Syndrome (RHS, OMIM 129400). There is good genotype–phenotype correlation in p63 mutation-associated syndromes. For examples, p63 mutations associated with EEC syndrome are exclusively found in the DNA-binding domain of the protein, and those associated with AEC or RHS syndrome are located either at the N-terminal TA^ΔN^ domain or C-terminal TID or SAM domain of the ΔNp63α isoform [5] (Fig. 1).

Among these disorders, EEC syndrome is the prototype of p63 mutation-associated disorders, as it exhibits defects of all three hallmarks. About 30% of EEC patients have skin defects, and the skin is often thin and dry and sometimes resembles dermatitis. Other affected tissues with ectodermal origin include hair, teeth, nails, and lacrimal ducts (Table 1). These phenotypes are reminiscent of those of p63 ΔN-specific or complete knockout mouse models [5], although in less severe forms. More than 90% of EEC syndrome patients carry heterozygous mutations in the p63 DNA-binding domain with a few hot spots of amino acids residues R204, R227, R279, R280, and R304. Transient transfection assays and in vitro DNA-binding assays have shown that these mutations disrupt p63 binding to DNA and act in a dominant negative fashion [36–38]. Two other syndromes with p63 mutations in the DNA-binding domain are LMS and ADULT syndromes. Both syndromes have mammary gland hypoplasia (Table 1). However, LMS patients rarely have defects in their skin and hair phenotypes, whereas ADULT patients have skin, nail and teeth defects. LMS mutations are also found in N-terminal TA^ΔN^ and C-terminal SAM domains of p63. For ADULT syndrome, the amino acid R298 in the DNA-binding domain is the most significant hot spot of mutations [39]. Rather than the dominant negative model that is proposed for EEC mutations, R298 mutations implicated in ADULT syndrome are proposed to be gain-of-function [36]. Another reported ADULT syndrome mutation is N6H, located in the ΔN-specific domain of p63 (Fig. 1) [5].

**Table 1:** Phenotypes of skin and ectodermal-derived appendages in p63 mutation-associated syndromes

	Skin	Ectodermal-derived appendages
EEC syndrome	Mild phenotype—dry and thin skin	Hair, nails, teeth and glands defects- highly variable severity
Limb mammary syndrome (LMS)	Rarely detected	Nails and lacrimal ducts defects, hypohydrosis, nipple hypoplasia and orofacial clefting
ADULT syndrome	Dry skin—milder than EEC	Teeth, nail and lacrimal duct defects
AEC syndrome	Severe skin erosions	Eyelid fusion, teeth and hair defects and/or alopecia, lacrimal duct obstruction and orofacial clefting
Rapp-Hodgkin syndrome (RHS)	Severe skin erosions	Teeth and hair defects and/or alopecia, lacrimal duct obstruction and orofacial clefting

Among all p63 mutation-associated syndromes, the most severe skin phenotypes are observed in AEC/RHS syndromes (Table 1). About 70–75% patients have severe skin erosions that sometimes resemble second-degree burns [40]. Defects of nails and teeth are also common phenotypes in AEC/RHS patients. In general, the ectodermal dysplasia phenotypes are milder in RHS, compared to AEC. Mutations involved in AEC/RHS syndromes, either in the N- or C-terminal region of the ΔNp63α, have been shown to disrupt the transactivation activity of the ΔNp63α isoform in a dominant fashion.

It is generally believed that dominant negative or gain-of-function is the disease mechanism of *TP63* mutations. This notion is supported by several lines of evidence. First, *TP63* mutations associated with human syndromes are heterozygous, and mostly missense mutations [5]. In a disease case where the complete *TP63* gene was lost in a larger genomic deletion region, the patient did not present any ectoderm dysplasia phenotype [41]. Second, studies using in vitro biochemical methods, such as transient transfection, DNA-binding assays and molecular structure modeling demonstrated that mutant p63 has dominant negative or gain-of-function effect towards the wild-type p63 [36, 38, 39]. Third, heterozygous p63 knockout mouse models do not exhibit any epidermal phenotype. To model the dominant negative or gain-of-function effect of *TP63* mutations in human disease, two knock-in mouse models carrying Trp63 (encoding p63 in mice) mutations recapitulating EEC (R279H in human) [42] and AEC (L514F in human) [43] were reported. In the EEC knock-in model, one mouse allele carried a floxed neomycin cassette, and is herein referred to as Trp63^R279HN^. Heterozygous Trp63^R279HN^ mice exhibited phenotypes that are similar to those observed in EEC syndrome patients. This includes cleft palate, anomalies of the distal limbs, defective tooth morphogenesis, and dystrophic nails. Although no major skin abnormalities were detected in Trp63^R279HN^ mice, primary keratinocytes from these mice exhibit reduced proliferation and increased senescence. Furthermore, an increased level of p63 mRNA and protein was detected in Trp63^R279HN^ mice, which is consistent with observations reported in the epidermis of p63 EEC patients [38]. The heterozygous AEC L514F mouse model presents hypoplastic and fragile skin, ectodermal dysplasia and cleft palate, resembling defects observed in AEC syndrome patients [5]. These phenotypes probably result from impaired FGF signaling, as two p63 direct target genes Fgfr2 and Fgfr3 showed reduced expression [43].

## p63-dependent gene regulation in epidermal keratinocytes

Due to the striking epidermal phenotype of p63 knockout mouse models and of p63 mutation-associated human diseases, the molecular and cellular role of p63 in epidermal keratinocytes has been extensively studied. It has been shown that p63 is important in both keratinocyte proliferation and differentiation [10, 44]. Higher p63 expression level is found in more proliferative cells, such as holoclones that represent epidermal stem cells [9], indicating that p63 is important for keratinocyte proliferation. Many studies have used siRNA knock-down approaches to investigate the role of p63 in proliferation and differentiation. Knock-down of p63 in primary keratinocytes gives rise to hypoplasia, hypoproliferation, cell cycle arrest [10] and abnormalities in cell adhesion [8]. Furthermore, downregulation of p63 prevents cells from differentiating and stratifying in both 2D and 3D keratinocyte differentiation models [10]. Although proliferation and differentiation are related, p63 seems to have independent roles in both processes. This was supported by a study where p63 knock-down caused both hypoplasia and differentiation defects, and concomitant p53 knock-down can rescue only the hypoplasia deficiency but not differentiation defects [10]. At the molecular level, knock-down of p63 induces genes that control cell cycle arrest, such as p21 (CDKN1A) [10, 45, 46], and genes that negatively regulate cell proliferation, such as JunB [47]. At the same time, knock-down of p63 downregulates genes that can positively regulate cell proliferation, such as Fos and c-Jun [47], and genes that are important for epidermal differentiation, such as Perp and K14 [48, 49]. Consistently, many genes that are involved in cell proliferation, cell cycle control, and keratinocyte differentiation have been shown to be direct target genes of p63 (see review [50]). In addition to controlling cell cycle genes, p63 is shown to directly regulate genes involved in glycolytic metabolism in human keratinocytes, such as hexokinase 2 (HK2) and 6-phosphofructo-2-kinase/fructose-2,6-bisphosphatase 3 (PFKFB3) [51, 52]. HK2 phosphorylates glucose to produce glucose-6-phosphate, representing the first rate-limiting step in glucose metabolism pathways [53]. PFKFB3 is a key regulator that promotes glycolysis. Downregulation of p63 resulted in reduced expression of HK2 and PFKFB3 and concomitant decrease of glycolysis and cell proliferation. These data showed that p63 plays a role in keratinocyte metabolism, a novel mechanism to maintain high proliferation in keratinocytes [54].

The identification of p63 target genes has been a major effort in the field, either through dedicated candidate gene studies or through genome-wide explorative approaches. These studies identify the genomic binding regions of p63 and of p63-regulated genes most often by either knock-down or overexpression of p63 [47, 55], as discussed previously in this review. Some target genes are identified in the context of p63 mutation-associated diseases [56]. It should be noted that p63 binding in human and mouse keratinocytes shares only limited conservation [57]. Epidermal development-related genes seem to be regulated by conserved p63-bound genomic regions, whereas divergent p63-bound regions are involved in metabolic pathways. The distinct regulation controlled by p63 in human and mouse keratinocytes probably reflects the anatomical differences between human and mouse skin.

As a master regulator of epidermal development, p63 regulates transcriptional programs not only through its target genes but also via higher order regulation. In recent years, a number of studies have shown that p63 plays a role in epigenetic regulation in epidermal keratinocytes via several independent mechanisms. p63 can recruit HDAC1/2 to control the repression of cell cycle arrest genes, such as p21 and 14-3-3σ [46]. p63 was shown to be a repressor rather than an activator for cell cycle arrest genes, consistent with positive regulation controlled by p63 in keratinocyte proliferation. Furthermore, several chromatin factors are direct p63 target genes [11–13]. Among these factors, SATB1 regulates large-scale chromatin remodeling in specific cell types [58, 59]. In keratinocytes, SATB1 binds to the Epidermal Differentiation Complex (EDC) locus and compresses the conformation of EDC where many epidermal terminal differentiation genes are located [11]. In mice, overexpression of Satb1 can partially rescue the phenotype of p63-deficient mouse skin [11]. Another p63 direct target, BRG1, is an ATP-dependent chromatin remodeler. In keratinocytes, BRG1 promotes relocation of the EDC locus from the nuclear periphery towards nuclear interior, which is associated with upregulation of genes in the EDC locus [12]. A chromobox protein, CBX4, that is a component of Polycomb Repressive Complex 1 (PRC1), is also regulated directly by p63 [13]. CBX4 seems to mediate the p63-dependent repression of non-epithelial genes, and overexpression of CBX4 can rescue p63 deficiency in keratinocytes. Chromatin remodelers that are direct p63 targets also include LSH, which regulates DNA methylation and transcriptional silencing [60]. LSH seems to act in p63-mediated senescence. Recently, p63 was shown to regulate the expression of nuclear envelop-associated components (Lamin B1, Lamin A/C, SUN1, Nesprin-3, Plectin) [16]. Several nuclear shape-associated genes, such as Sun1, Syne3 and Plec, were shown to be p63 direct targets. In the epidermis of p63 complete knockout mice, these genes were downregulated, and skin epithelial cells displayed an altered nuclear shape. Consistently, keratinocyte-specific gene loci were relocated away from sites of active transcription toward the heterochromatin-enriched repressive nuclear compartments in p63 depleted cells.

**Fig. 2:**
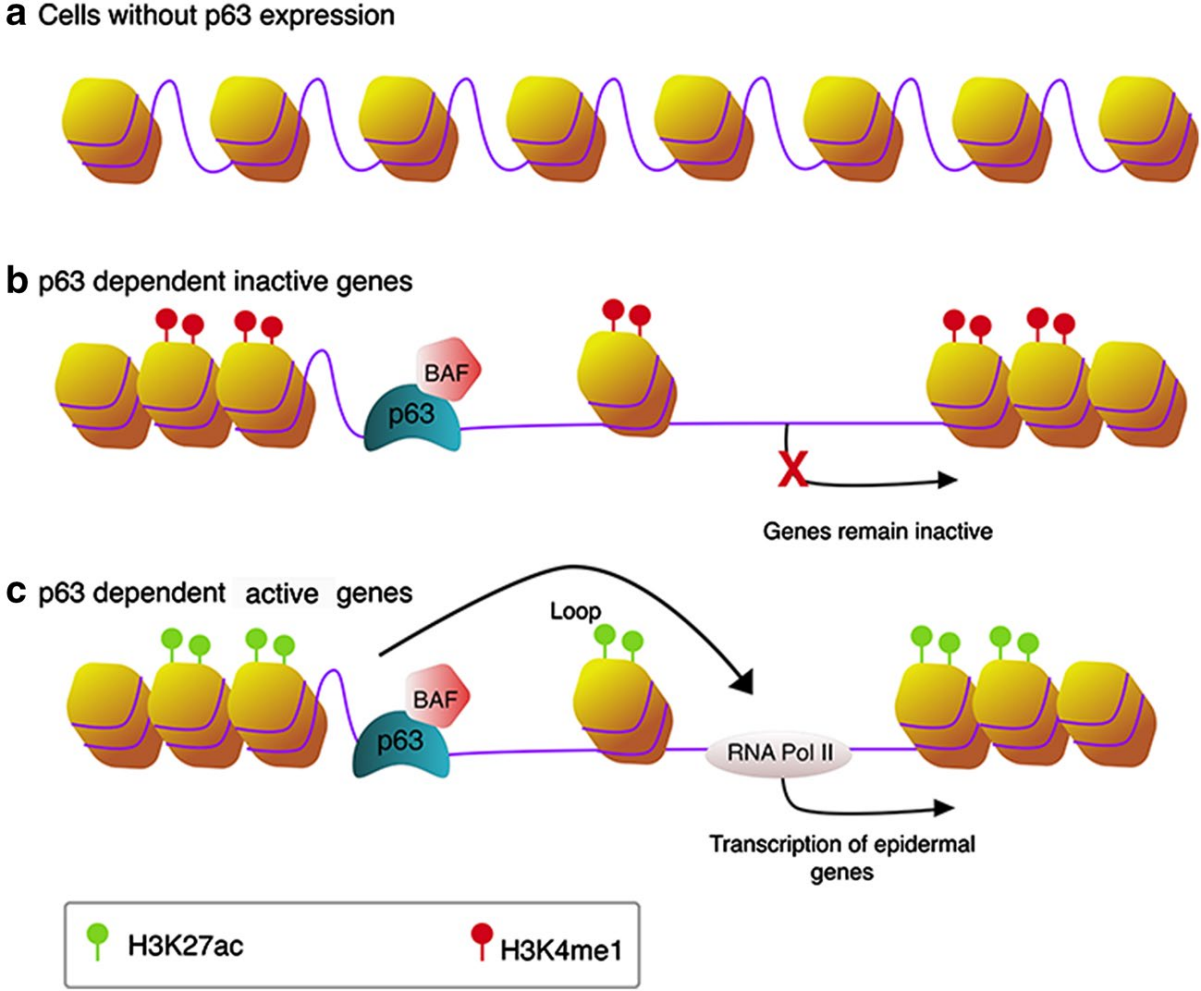
The bookmarking role of p63 in epithelial cells. **a** In the absence of p63, either in embryonic stem cells before epidermal commitment or in non-epithelial somatic cells, loci of p63-dependent genes remain closed and are occupied by nucleosomes. **b**, **c** In epithelial cells where p63 is expressed, p63 and co-regulators such as BAF can engage closed chromatin and promote its opening. In specific (embryonic) epithelial cell types, some genomic loci are bookmarked by p63 but nearby genes are not activated by p63. These open chromatin sites are marked by H3K4me1 (**b**). Other loci and genes are bookmarked and activated by p63. These regions are marked by active enhancer mark H3K27ac (**c**)

In addition to regulating chromatin factors and nuclear envelop-associated components involved in higher order gene regulation in keratinocytes, it has become evident that p63 plays a master role in regulating enhancers. It should be noted that many studies discussed below took unbiased genomic approaches rather than p63-centered approaches, yet they came to the conclusion that p63 is the most significant transcription factor that controls epidermal enhancers. Bao et al. showed that genomic regions of p63-bound enhancers detected in keratinocytes are nucleosome-enriched and inaccessible in cells where p63 is not expressed (Fig. 2a), and the cooperation of p63 and BAF is required for opening chromatin (Fig. 2b, c) [61]. This suggests that p63 and probably its co-regulators have a pioneer factor-like function during epidermal development. Pioneer factors are a class of transcription factors that are the first to engage compact chromatin and to create an accessible chromatin and epigenetic environment for other TFs to be recruited to enhancers [62–64]. They bind to target DNA sites before the onset of transcription, and often pioneer factor-bound regions are open chromatin regions in cells where these factors are expressed and are otherwise closed in cells where they are not expressed. Pioneer factors have been shown to be important for cell identity and commitment. Consistent with this notion, another study on p53 reported that, in addition to binding to the usual active promoters and enhancers of p53-regulated genes, p53 can also bind to inaccessible regions of the chromatin in lung fibroblasts [65]. Interestingly, these inactive regions in lung fibroblasts are seemingly active in two epithelial cells, normal human epithelial keratinocytes (NHEK) and human mammary epithelial cells (HMEC), but are repressed in other cell types. As p63 and p53 share high sequence and structure homology in their DNA-binding domains, and they bind to essentially the same binding motifs on DNA, it has been argued that these ‘proto-enhancers’ bound by p53 can be active enhancers regulated by p63 or by both p53 and p63 in epithelial cells. Therefore, p53 and p63 may function as pioneer factors. In agreement, epigenomic profiling analyses by ChIP-seq of histone enhancer marks and p63 [14, 29] and by mapping of DNA (hydroxy)methylation sites [15] during keratinocyte differentiation showed that epidermal enhancers are most significantly enriched for p63-binding motifs and that p63 binds mainly to enhancers. p63-bound enhancers are not always active in regulating nearby genes (Fig. 2b). It has been proposed that these p63-bound genomic loci are bookmarked by p63. In particular epithelial cell types or at developmental stages, p63 cooperates with specific transcription factors to activate gene expression in a cell type- and developmental stage-specific manner (Fig. 2c) [14, 50]. Taken together, these observations support an appealing model where p63, probably together with co-regulating transcription and chromatin factors, such as BAF [61], acts as a pioneer factor to open chromatin in epithelial cells and shapes the enhancer landscape.

In summary, p63 is likely to regulate gene expression in epidermal keratinocytes at several levels: it regulates direct target genes involved in keratinocyte proliferation and differentiation; it directly regulates chromatin and epigenetic factors; and it might act as a pioneer factor to shape the chromatin and enhancer landscape, and thereby regulate global gene expression.

## p63 is essential for proper epidermal commitment

Consistent with the recent proposed pioneer role of p63 in epidermal development, classical studies in mice nearly two decades ago showed that p63 is essential for proper epidermal commitment [1, 3]. Two different groups reported that mouse models lacking p63 show striking defects of the epidermis including either partial [1] or complete [3] absence of stratified epithelia. During early vertebrate development, the surface epithelium is composed of a single layer of K8/K18-positive ectodermal cells [66]. This single-layered epithelia is functionally important for cellular diffusion, secretion or absorption, but not directly relevant for barrier function [67]. As development proceeds, these ectodermal cells develop into keratinocytes that have high expression of mature epithelial markers, such as K5/K14 and can further develop to the epidermis that has a barrier function. This physiological transition is called epidermal commitment. During this process, proper p63 expression and function is essential for correct commitment by activating epidermal genes and repressing genes of other lineages, and therefore p63 has been proposed to be the ‘gatekeeper’ of the epithelial lineage [68]. Studies using in vivo and in vitro models and p63-regulated genes are discussed here.

Recently, spatio-temporal expression of p63 during normal mouse embryonic development was analyzed [26]. p63 expression was detected in ectodermal cells near the newly formed somites and the posterior part of the E8.5 embryo, preceding epidermal commitment. Subsequently, p63 expression was enriched at branchial arches and the limb buds, and then expanded to the whole surface of the embryo. The embryonic expression pattern of p63 correlates remarkably well with affected structures, such as the epidermis, orofacial regions and limbs in p63 knockout mice and in p63 mutation-associated human diseases. In normal mice, the single-layered epithelium eventually develops into stratified epithelium, whereas the surface ectoderm of p63 null mouse strains remains a monolayer of non-proliferating cells expressing K8/K18 [68]. Interestingly, genetic complementation with ∆Np63 in p63 null mice rescues the development of the skin through activation of epidermal genes, such as the direct p63 target K14 [69], emphasizing the important role of ∆Np63 in epidermal development. Consistently, in vitro studies using mouse embryonic stem cells (mESCs) showed that p63 expression is detected before expression of the epidermal marker K14 [70–73]. p63 deficiency in mESCs impedes progression towards the stratified epithelial fate, and p63-deficient cells have abnormally upregulated mesodermal genes [6, 73], indicating loss of the epithelial cell fate. Furthermore, ectopic expression of ΔNp63 in mESC-derived p63-deficient ectodermal cells can induce epidermal commitment and lead to differentiation into keratinocytes [6, 70]. It has become evident that p63 induces expression of many epidermal genes, such as K14, K17 and S100A11, by directly binding to these gene loci [68]. However, it is also important to investigate whether repressed expression of genes from other lineages is directly regulated by p63 or through p63-related higher order regulation. In summary, these in vivo and in vitro studies support the master regulatory role of p63 in epidermal commitment.

Although the essential role of p63 in epidermal commitment has been established, the gene networks and regulatory mechanisms of epidermal commitment that are upstream and downstream of p63 are not yet fully understood. A closer look into molecular pathways during epidermal development reveals the interaction of p63 and bone morphogenetic protein (BMP) signaling [74]. The ΔNp63 isoform activates BMP signaling through direct binding in conserved regulatory regions on the Smad7 promoter, thereby repressing its expression. p63 sustains Bmp7 expression in the epidermis and indirectly controls Bmp4 expression in the dermis. Equally important, during early embryonic development, BMP signaling acts as an epidermal inducer by suppressing the neural fate, probably via p63-regulated gene expression [66, 75, 76]. Furthermore, p63 expression is regulated in a positive autoregulatory manner through a long-range enhancer (p63LRE) [77]. This enhancer is conserved in both human and mouse and is also bound by several other transcription factors including AP2, Cebpa, Cebpb and the POU domain-containing protein Pou3f1. Among them, Cebpa, Cebpb and Pou3f1 repress p63 expression [77]. It has also been reported that Cebpb binds to the p63 promoter to repress its expression [78]. These studies help unravel the p63-related regulatory network controlling epidermal cell fate. However, continuing efforts to comprehensively and systematically identify p63-dependent transcriptional networks are warranted.

Taken together, in vivo and in vitro studies have demonstrated that p63 is essential for epidermal commitment. The molecular pathways and gene regulatory mechanisms that act during epidermal commitment remain to be elucidated. It will be particularly interesting to investigate whether p63 functions as a pioneer factor during this process, either alone or most probably together with other co-regulators, and how p63 mutations affect this process.

## p63 in cellular reprogramming

**Fig. 3:**
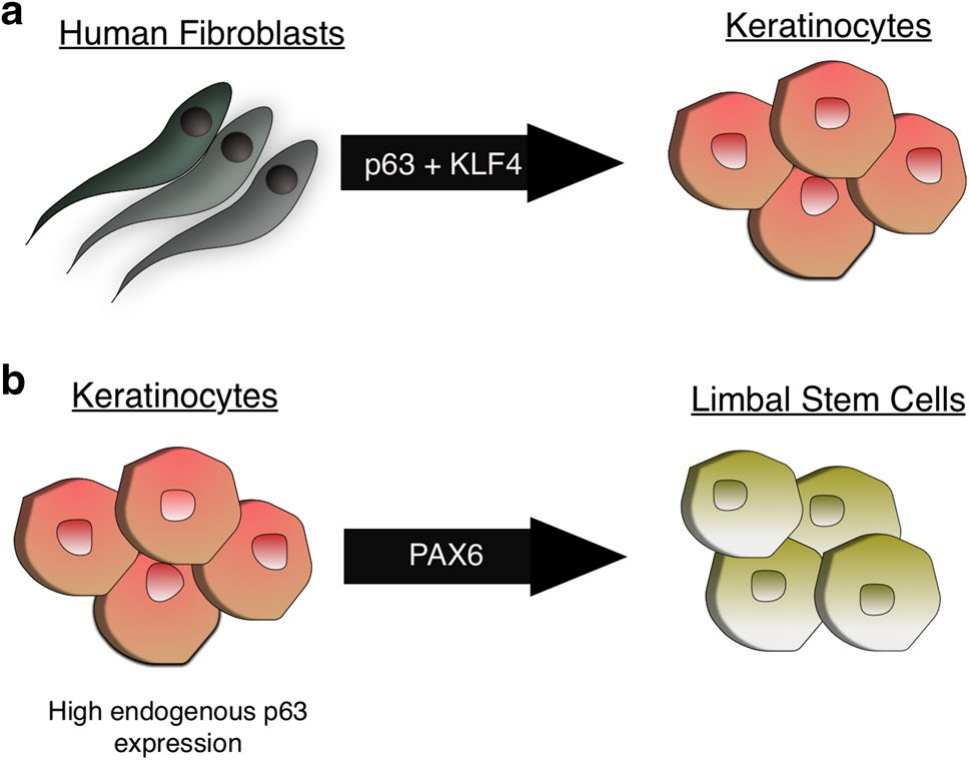
p63-dependent transdifferentiation. **a** Human fibroblasts can be reprogrammed into Keratinocyte-like cells through ectopic expression of p63 and KLF4. **b** Keratinocytes that have high endogenous p63 expression can be reprogrammed into limbal stem cells by ectopic PAX6 expression

Somatic cell transdifferentiation is a process in which transient ectopic expression of lineage master regulators can induce conversion into a different somatic cell fate without passing through a pluripotent configuration [79–81]. Given the master regulatory role of p63 for proper epidermal commitment and maintenance, hypotheses have arisen that its ectopic expression in non-epithelial cell types might be sufficient to convert these cells to the epidermal fate. This has been demonstrated in a report where the combination of KLF4 with ΔNp63 could induce the conversion of human fibroblasts to keratinocyte-like cells [82] (Fig. 3a). The transdifferentiated cells showed positive staining for the basal marker K14 and were negative for fibroblast markers, such as MME and COL11A1. In the same study, cancer cells could also be converted to keratinocytes using KLF4 and p63, suggesting that these two transcription factors are capable of reprogramming cells to the epidermal fate even in different epigenetic backgrounds. Another example is the cooperation between p63 and PAX6 in driving limbal stem cell (LSC) commitment [83] (Fig. 3b). p63 is expressed in both epidermal keratinocytes and LSCs, whereas PAX6 is specific for LSCs. Epidermal keratinocytes and LSCs undergo distinct transcriptional programs during terminal differentiation, with an induction of K1/10 or K3/12 expression for epidermal keratinocytes or LSCs, respectively. Ouyang et al. showed that p63 was essential for both epidermal keratinocyte and LSC fates, and the combination of p63 and PAX6 defined the LSC fate, consistent with expression patterns of these two genes. Interestingly, LSCs with knock-down of PAX6 acquired the epidermal keratinocyte property of K1/K10 induction during terminal differentiation; in contrast, ectopic expression of PAX6 in epidermal keratinocytes gave rise to LSC-like cells. This report is in line with previous studies where reported mutations in either in PAX6 [84] or p63 [85] lead to blindness resulting from limbal stem cell deficiency.

Two studies have also suggested that p63 plays a role in cell reprogramming to a pluripotent cell state. In one study, the authors reported that p63 enables reprogramming to pluripotency in mouse embryonic fibroblasts [86]. In this case, p63 did not induce reprogramming per se, but was important for maximal reprogramming efficiency. On the contrary, another report showed that p63 knockout mouse keratinocytes acquired some pluripotent signatures [87], suggesting that p63 blocks reprogramming to pluripotency. As p63 is normally not expressed in pluripotent stem cells, the function of p63 in pluripotency is completely unknown. Whether and how p63 plays a role in cell reprogramming to pluripotency requires further investigation.

So far, gene regulatory programs during these cell fate conversions have not yet been identified. The precise role of p63 in gene regulation during cellular reprogramming, especially towards an epithelial fate or during a switch between different epithelial fates, is important for understanding its master regulatory function controlling epithelial cell identity. Beyond exploring the fundamental biological role of p63, the examples discussed in this review also hold great potential for developing therapeutic options, e.g. converting human epidermal keratinocytes to LSCs for cornea regeneration or to other rare epithelial types.

## Potential therapeutic approaches

Other than cosmetic surgery and correction [88], there is no cure for p63 mutation-associated disorders. Recent improvements in modeling human diseases have opened new perspectives on amelioration of these diseases.

**Fig. 4:**
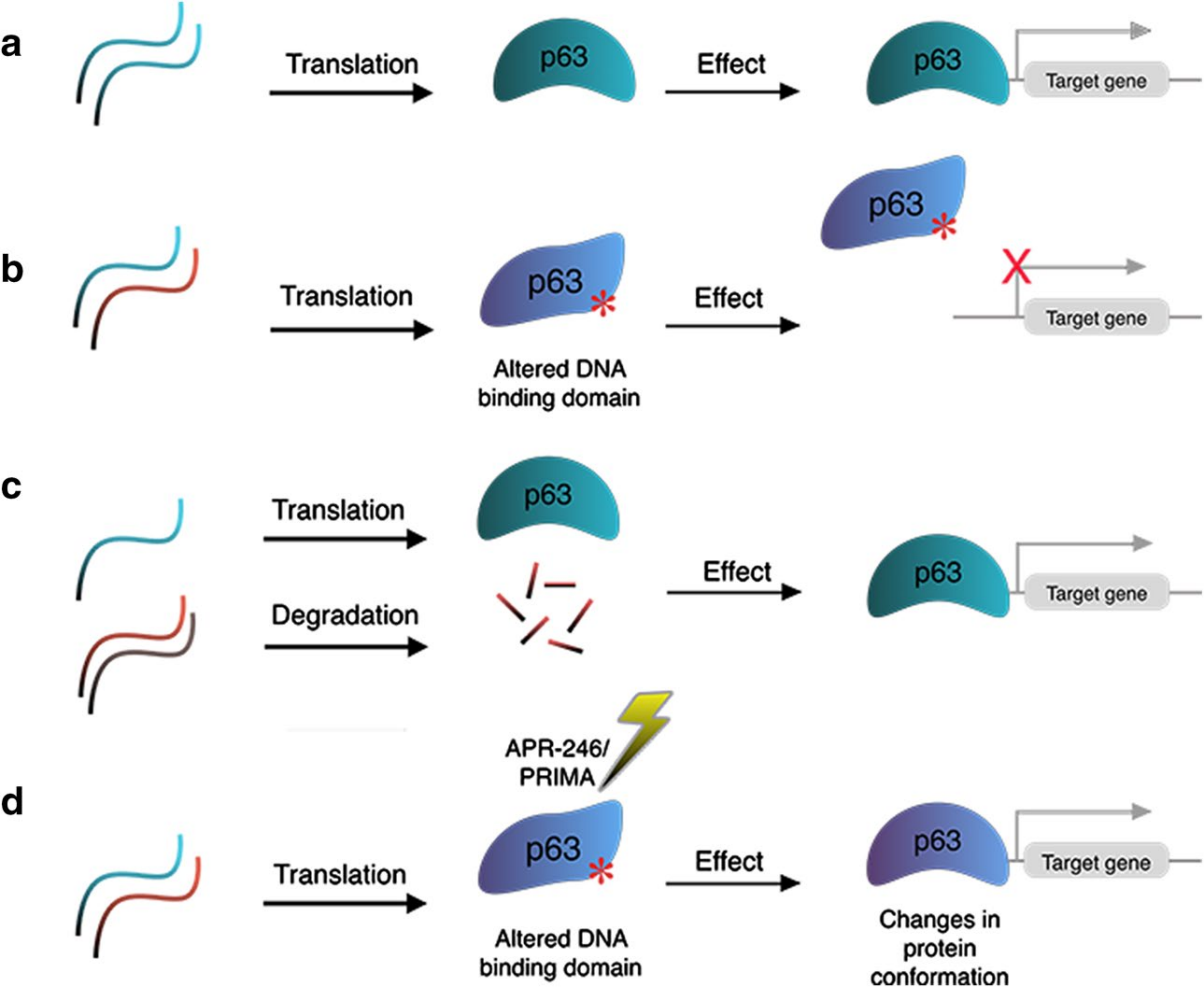
Potential therapeutic approaches targeting p63 mutations. **a** In normal epidermal cells, p63 can bind to promoter and enhancer regions in the genome and regulate expression of its target genes. **b** Mutations in the DNA-binding domain of p63 (EEC syndrome mutations) disrupt p63 DNA-binding, thereby deregulating its target genes. **c** Silencing the mutant allele of EEC syndrome mutations by mutation-specific siRNAs allows wild-type p63 binding and activation of p63 target genes. **d** The use of the small molecular compound APR-246/PRIMA-1^MET1^ possibly changes the conformation of mutant p63 protein and restores its ability to bind and activate its target genes

Initially designed to treat cancers, the small compound APR-246/PRIMA-1^MET^ was shown to interact with the mutant p53 core domain and promote its folding to the wild-type conformation [89, 90]. This compound has been tested in a phase I/II clinical trial in patients with either hematological malignancies or hormone-refractory prostate cancer [91]. Given the high degree of homology in the DNA-binding domain between p53 and p63 [92], the potential of APR-246 for restoring mutant p63 conformation and function was tested in two cellular models: epidermal stratification and corneal commitment [73, 93]. Shen et al. reported that APR-246 rescued differentiation defects of keratinocytes established from EEC patients carrying p63 mutations R204W and R304W in the DNA-binding domain, specifically in terms of cell morphology and gene expression in 2D and 3D epidermal stratification models [93]. A set of epidermal differentiation markers, such as K10, TGM, CysME, LCE2, and Filaggrin, were efficiently induced in APR-246-treated p63 mutant keratinocytes, approaching similar expression levels as observed in wild-type p63 keratinocytes. In addition, p63 target genes, such as ADH7 and Claudin-1, which were downregulated in p63 mutant keratinocytes, were upregulated upon APR-246 treatment. Another report using human-induced pluripotent stem cells (hiPSCs) established from the same EEC patients showed corneal epithelial cells derived from these patient hiPSCs responded to APR-246, and impaired corneal epithelial differentiation was rescued [73]. As p63 EEC mutations likely have a dominant negative effect towards the wild-type protein (Fig. 4a, b), APR-246 can probably restore mutant p63 to the wild-type conformation, thereby rescuing p63 DNA-binding and target gene activation (Fig. 4c). Further in-depth cellular and molecular analyses, such as RNA-seq and ChIP-seq, should be performed to comprehensively evaluate the effect and underlying mechanism of APR-246 on cell differentiation. Ultimately, in vivo studies and clinical trials are necessary to explore the therapeutic potential of APR-246.

In addition to small compounds, small interfering RNAs (siRNA) have also been explored for their therapeutic potential in treating p63 mutation-associated diseases. Novelli et al. reported the identification of a panel of siRNAs specifically targeting the p63 EEC R304W mutant transcripts. These R304W-targeting siRNAs can rescue defects during corneal epithelial differentiation of hiPSCs carrying the R304W mutation [94]. Similarly, the siRNA approach has also been applied to specifically target the p63 EEC R279H transcripts in oral mucosa epithelial stem cells (OMESCs) from EEC patients. Treatment with these siRNAs restored the epithelial differentiation capacity of these patient cells [95]. The success of these siRNA approaches also confirms the dominant negative model of p63 EEC mutations and demonstrates that degrading the mutant allele alone is sufficient to rescue the epithelial defects caused by these mutations (Fig. 4d).

In addition to approaches discussed in this review, rapidly emerging technological tools, such as genome editing, have provided new possibilities for developing therapeutic options for p63 mutation-associated diseases. Further in vivo studies and clinical trials are necessary to validate these therapeutic options and eventually to treat patients.

## Conclusions and future perspectives

Since the discovery of the transcription factor p63 nearly two decades ago, its master regulatory role in the development of the epidermis has been extensively studied in cellular and animal models and at normal and diseased states. Although many isoforms have been identified for the p63 protein, the ΔNp63α is the most abundant isoform in epidermal cells and is probably responsible for the bulk of the functional p63 activity. p63 plays its key role in epidermal commitment and terminal differentiation, not only by regulating its direct target genes, but also by shaping the chromatin state, in particular, the enhancer landscape. Recent in-depth molecular analyses suggest that p63 functions as one of the pioneer factors for epidermal cell identity. This model is supported by classical cellular and in vivo studies as well as recent transdifferentiation studies where p63 is required for epithelial cell lineages. Testing this pioneer factor model during epidermal commitment at the molecular level and identifying key regulators that cooperate with p63 are critical topics for future research. It is plausible that p63 mutations involved in human disorders may deregulate gene expression by disrupting the pioneer factor function and thereby the epithelial epigenetic landscape, subsequently giving rise to epidermal phenotypes. Current and ongoing efforts to understand the role of p63 in normal and diseased conditions are not only important for the fundamental insights into gene regulation and epidermal development, but also for the development of novel therapeutic strategies to treat patients with p63 mutation-associated and other related diseases. Although therapeutic development for these diseases is still at its infancy, encouraging results of small molecule compounds and siRNA approaches have opened new possibilities, and should be extended further to eventually benefit patients.
